# A Single-Plasmid Genome Editing System for Metabolic Engineering of *Lactobacillus casei*

**DOI:** 10.3389/fmicb.2018.03024

**Published:** 2018-12-05

**Authors:** Yongping Xin, Tingting Guo, Yingli Mu, Jian Kong

**Affiliations:** State Key Laboratory of Microbial Technology, Shandong University, Qingdao, China

**Keywords:** *Lactobacillus casei*, acetoin, NICE system, lactose operon, metabolic engineering

## Abstract

Genome engineering of *Lactobacillus casei*, an important industrial microorganism for dairy fermented product, currently relies on inefficient and time-consuming double crossover events. In this study, we developed an easy-to-use genome engineering strategy for metabolic engineering of *L. casei* for acetoin production. Plasmid pMSP456-Cre, that contains prophage recombinase operon *LCABL_13040-50-60* driven by the nisin-controlled inducible expression (NICE) system and the site-specific recombinase gene *cre* under the control of the promoter of the lactose operon from *L. casei*, was constructed. Using this plasmid, integration of a *hicD3* gene linear donor cassette (up-*lox66*-cat-*lox71*-down) was catalyzed by the LCABL_13040-50-60 recombinase and the *cat* gene was excised by the Cre/lox system with an efficiency of 60%. To demonstrate this system for sequential and iterative knocking out genes in *L. casei*, another three genes (*pflB, ldh* and *pdhC*) related to acetoin production were deleted with the efficiencies of 60, 40, and 60%, respectively. The yielding quadruple mutant could produce a ∼18-fold higher amount of acetoin than the wild-type and converted 59.8% of glucose to acetoin in aerobic. Therefore, these results proved this simple genome engineering strategy have potential in metabolic engineering of *L. casei* for production of high value-added metabolites.

## Introduction

Lactic acid bacteria (LAB), largely from the order Lactobacillales, are native to food-related habitats and long historical used for the production of fermented beverages and foods (Duar et al., 2017; Bron and Kleerebezem, 2018). Nowadays, with the advent of next-generation whole genome sequencing and functional genomics, abundant knowledge about LABs are available, including their metabolic pathway which offered the possibility to engineer LAB genomes to serve as cell factories (Zhu et al., 2017). Therefore, to development of easy-to-use genome editing tools for LABs to make full use of these genomics data to design probiotic strains with desired functions is very instant (Hidalgo-Cantabrana et al., 2017; Yadav et al., 2018).

*Lactobacillus casei*, as an important dairy industrial LAB strain, also plays potential roles in the medical and pharmaceutical fields, such as reducing 1,2-dimethylhydrazine-associated colorectal cancer (Lenoir et al., 2016), and is receiving more attention for production of high-valued metabolites, such as acetoin (3-hydroxy-2-butanone or acetyl methyl carbinol) (Nadal et al., 2009; Reale et al., 2016a,b; Bosma et al., 2017), a flavoring compound naturally occurs in wine, honey, milk, coffee, fresh strawberry, etc. (Sun et al., 2012; Zhang et al., 2013). However, due to the inefficient and laborious genome engineering tools which relied on the double crossover events (Munoz-Provencio et al., 2012; Song et al., 2014), the number of highly and satisfactorily engineered *L. casei* strains for biotechnological production of acetoin is still relatively low.

The recently reported tool for editing *L. casei* genomes was the CRISPR-Cas9^D10A^ nickase-assisted homologous double-crossover method which was able to mediate deletion of 3 kb in the proportion of 66% (Song et al., 2017). However, to cure the plasmid for sequential genome editing, the recombinants containing editing plasmids need to be streaked on MRS plates without erythromycin for about 2–3 times (Song et al., 2017). Most recently, a two-plasmids system (pMSP456 and pMSPCre) based high-efficiency genome editing tool has been established for construction of deletion mutants in our previous study (Xin et al., 2017). Plasmid pMSP456 was used for expression the LCABL_13040-50-60 recombinases (a presumptive 5′-3′ exonuclease LCABL_13060, a ssDNA annealing protein LCABL_13050 and a predicted host nuclease inhibitor LCABL_13040 analogous to Exo, Beta and Gam of the λ Red system from λ phage (Yu et al., 2000), respectively. LCABL_13040-50-60 recombinases could mediate integration of a linear donor cassette (up-*lox66*-cat-*lox71*-down) into the targeted gene (Sternberg and Hamilton, 1981). After curing the plasmid pMSP456, the plasmid pMSPCre which was used for expression of Cre recombinase should be transformed into the recombinant clones which integrated with the *lox66*-cat-*lox71* cassette. The Cre recombinase could excise the *cat* gene through the two mutant *lox* sites (*lox66* and *lox71*) in which the 8 bp core sequence in same orientation, leaving a *lox72* site which displays strongly reduced recombination ability mediated by Cre recombinase (Sternberg and Hamilton, 1981; Lambert et al., 2007). However, it is too time-consuming to cure these two plasmids and retransformation of them for sequential genes deletion (Xin et al., 2017). Therefore, a single-plasmid which contains prophage recombinases operon *LCABL_13040-50-60* and the site-specific recombinase gene *cre* under the control of two different inducible expression system is hopeful for easy-to-use genome editing in *L. casei*.

In LAB, including *L. casei*, one of the most commonly used inducible expression system was the nisin-controlled inducible expression (NICE) system from *Lactococcus lactis* (Kleerebezem et al., 1997; Eichenbaum et al., 1998; Mierau and Kleerebezem, 2005). In this system, expression of *nisA* and *nisF* in the nisin cluster is controlled by the two-component regulatory system including a response regulator NisR and a sensor kinase NisK (Kuipers et al., 1995; de Ruyter et al., 1996). When addition of extracellular nisin, NisK would phosphorylate NisR, subsequently activates the expression of the promoter of *nisA* (Kuipers et al., 1995; de Ruyter et al., 1996). The other well-characterized system for inducible expressing foreign genes was based on the promoter of the lactose operon *lacTEGF* from *L. casei* BL23 which regulated by very tight glucose repression and substrate induction mechanisms and made it a tempting candidate or the lactose-inducible expression of the site-specific recombinase Cre (Gosalbes et al., 2000).

In this study, a single-plasmid pMSP456-Cre, that contains prophage recombinases operon *LCABL_13040-50-60* driven by the NICE system and the site-specific recombinase Cre under the control of the promoter of the lactose operon from *L. casei*, was constructed. Using this single-plasmid system, four different genes (*hicD3, pflB, ldh*, and *pdhC*) responsible for acetoin biosynthesis were subsequently deleted to investigate the feasibility of high level of acetoin production, the aim of this study was to provide an easy-to-use genome editing tool for metabolic engineering in *L. casei*.

## Materials and Methods

### Bacterial Strains, Plasmids and Culture Conditions

The strains and plasmids used in this work are listed in Table 1. Unless otherwise stated, Lactobacilli and their derivatives were grown in deMan Rogosa Sharpe (MRS) broth (Oxoid) which is a rich medium with glucose as carbon source at 37°C under static conditions. As cloning hosts, *Escherichia coli* DH5α and XL1-Blue were grown aerobically in Luria-Bertani (LB) medium at 37°C. If necessary, antibiotics were supplemented as follows: 5 μg/ml erythromycin or chloramphenicol and 250 μg/ml erythromycin for *E. coli* XL1-Blue, 100 μg/ml ampicillin and 30 μg/ml kanamycin for *E. coli* DH5α, respectively. For inducible expression of the Cre recombinase, the sugar-free MRS medium (Zhang et al., 2016) supplemented with 2% lactose (LMRS) was used.

**Table 1:** Plasmids and bacterial strains used in this study.

Strain or plasmid	Characteristic(s)	Source
Strains		
*Escherichia coli*		
DH5α	Subcloning host	Novagen
XL1-Blue	Subcloning host	Novagen
*Lactobacillus casei*		
BL23	Wild-type	Maze et al., 2010
BL24	Derivative of *L. casei* BL23 (Δ*hicD3*)	This study
BL25	Derivative of *L. casei* BL23 (Δ*hicD3*, Δ*pflB*)	This study
BL26	Derivative of *L. casei* BL23 (Δ*hicD3*, Δ*pflB*, Δ*ldh2*)	This study
BL27	Derivative of *L. casei* BL23 (Δ*hicD3*, Δ*pflB*, Δ*ldh2*, Δ*pdhC*)	This study
Plasmids		
pUC19	Amp^r^; cloning vector	Novagen
pET-28a	Kan^r^; cloning vector	Novagen
pMSP3535	Erm^r^	Bryan et al., 2000
pMSPcre	Source of *cre* gene	Xin et al., 2017
pUCgalK	Source of fragment *loxP*-*cat*-*loxP*	Xin et al., 2017
pMSP456-Cre	Expression LCABL_13040-50-60 under P_nisA_ control and Cre under P_lac_ control	This study


### DNA Manipulation

All the restriction endonucleases, DNA polymerases and T4 DNA ligase used in this study were purchased from TaKaRa (Japan). Amplification of the DNA fragments for cloning and dsDNA recombineering purposes were performed with 2 × Primestar Max while PCR amplification for screening purposes were generated by rTaq DNA polymerase. *E. coli* plasmids DNA were isolated by Plasmid Mini Kits (Omega) and the linear DNA fragments were purified by Gel Extraction Kits (Omega) or Cycle-Pure Kits (Omega). *L. casei* genomic DNA extraction was carried out using TIANamp Bacteria DNA kit (TIANGEN, China) after the cultures were subjected to lysozyme (50 mg/mL) treatment at 37°C for 1 h.

### Molecular Manipulation

**FIGURE 1:**
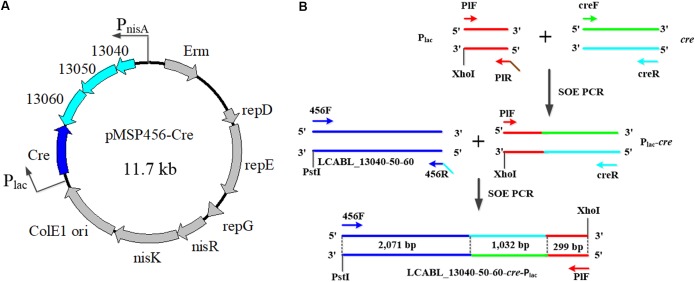
Scheme of plasmid pMSP456-Cre **(A)** and the construction of the cassette *LCABL_13040-50-60*-*cre*-P_lac_ **(B)**. 13040, 13050, and 13060 indicated the LCABL_13040, LCABL_13050 and LCABL_13060, respectively. P_nisA_, nisin promoter. P_lac_, lactose operon promoter. Erm, erythromycin resistance gene. SOE PCR, gene splicing by overlap extension PCR. NisR, a response regulator. NisK, a sensor kinase. *LCABL_13040-50-60* indicated the recombinase operon. PlF/PlR, creF/creR, 456F/456R refer to PCR primers and that the sequences are provided in Table 2.

Primers used in this study are listed in Table 2. The plasmid pMSP456-Cre (Figure 1A), which contains the operon of LCABL_13040-50-60 recombinases under the control of the nisin-inducible promoter P_nisA_ and a site-specific recombinase gene *cre* under the control of the lactose-inducible promoter P_lac_, was constructed from pMSP3535 (Bryan et al., 2000). The construction of the cassette *LCABL_13040-50-60*-*cre*-P_lac_ was shown in Figure 1B. *LCABL_13040-50-60* and the promoter P_lac_ of lactose operon were obtained from *L. casei* BL23 genomic DNA with primer pairs 456F/456R and PlF/PlR, respectively. *Cre* gene was generated from pMSPCre (Xin et al., 2017) using primers creF and creR. To obtain the cassette *LCABL_13040-50-60*-*cre*-P_lac_ (Figure 1B), the *LCABL_13040-50-60* and the fragment *cre*-P_lac_ which was generated by fusing the promoter P_lac_ and *cre* gene with primer pair PlF/creR were spliced by primers 456F and PlF. The cassette *LCABL_13040-50-60*-*cre*-P_lac_ was digested with PstI and XhoI and inserted into the compatible sites of pMSP3535 (Bryan et al., 2000), generating pMSP456-Cre.

**Table 2:** Oligonucleotide primers used in this study.

Primer	Sequence (5′-3′)	Restriction site
PlF	TATCTCGAGTAGCACTGATCATTAAAGAAC	XhoI
PlR	ACGGTCAGTAAATTGGACATGTTGTCATCACCTCCCAGTG	
creF	ACATGTCCAATTTACTGACCGTAC	
creR	TTAATCGCCATCTTCCAGCA	
456F	AACTGCAGATGACCATGCTTGATTACAACACAG	PstI
456R	TGCTGGAAGATGGCGATTAGCTACTCGACTAGCTCATCCATGCT	
catF	GAAAGATCTTACCGTTCGTATAATGTATGC	BglII
catR	GAAAGATCTTACCGTTCGTATAGCATACAT	BglII
catF1	CCGCTCGAGTACCGTTCGTATAATGTATGC	XhoI
catR1	CCGCTCGAGTACCGTTCGTATAGCATACAT	XhoI
catF2	ATCTGCAGTACCGTTCGTATAATGTATGC	PstI
catR2	ATCTGCAGTACCGTTCGTATAGCATACAT	PstI
hicD3-uF	AATGAATTCTGTTACGCAGAATGTTGACGG	EcoRI
hicD3-uR	GATCTAGAATAAAAAATCTCCTTTTCAAAATGC	XbaI
hicD3-dR	TATAAGCTTAGCGCATCATGGTTAAATCG	HindIII
hicD3-dF	ATAAAGCTTAAGTAGGTCCTTTTACGAGCG	HindIII
pflB-uF	GAACTCGAGTTTCTCGTGATGGCTACGTTAA	XhoI
pflB-uR	AGTTGTTTGCCTCCTAAAGTGG	
pflB-dR	CAAAAGCTTGTGTCTTTGAGGAAAAAATGCG	HindIII
pflB-dF	CCACTTTAGGAGGCAAACAACTAGATCTGCAGCTTAACTCAAGACAGGAA	BglII
ldh-uF	AATGAATTCTCGACAAAACTCATCGCTGC	EcoRI
ldh-uR	GTGATATCATCCTTTCTTATGTGC	
ldh-dF	GCACATAAGAAAGGATGATATCACCCTCGAGGACATCGAAACTCGTCAGTA	XhoI
ldh-dR	ATCAT GATCTAGAGTGACATAATTTGTAATGGCTTGG	XbaI
pdhC-uF	ACATGTCGACTTCCTAAAGGACACGTACGA	SalI
pdhC-uR	GTTGGCACCTCTTCTTTCTAG	
pdhC-dF	CTAGAAAGAAGAGGTGCCAACCTGCAGGCATGGTTGTAGGCGATTT	PstI
pdhC-dR	ACATGCATGCATCCCAGTTGTTGCAAGTTC	SphI


*The restriction sites in the primer sequences are underlined.*

The previously method was used for preparation of linear donor disruption cassette for recombineering (Xin et al., 2017). Briefly, the up–down fragment was generated by fusing the upstream and downstream homology arms which were obtained from the chromosomal DNA of *L. casei* BL23 and the chloramphenicol resistant *cat* gene flanked with the *lox66* and *lox71* sites was obtained by PCR from the plasmid pUCgalK (Xin et al., 2017) using primer pairs catF/catR, catF1/catR1 and catF2/catR2. The up–down fragment and *lox66*-*cat*-*lox71* fragment were inserted into the suitable sites of pUC19 or pET28a. The yielding vector was used for amplifying the linear donor disruption cassette for recombineering.

### The Feasibility of Single-Plasmid Genome Editing System in *L. casei* BL23

To demonstrate the single-plasmid genome editing system in *L. casei* BL23, we performed *hicD3* deletion. The recombineering steps were performed as previously reported (Xin et al., 2017). Briefly, expression of LCABL_13040-50-60 was induced by 5 ng/ml nisin (Sigma, United States) at initial OD_600_ of 0.25–0.30 until OD_600_ of 0.50–0.55 and preparation of electrocompetent cells (Yang et al., 2015). After electroporation, the mixture of linear donor double-strand DNA (dsDNA) disruption cassette (4 μg) and competent cells was kept on ice for 10 min. After electroporation (BioRad, 2000 V, 25 μF, 400 Ω), 1 mL SMRS (MRS supplemented with 0.1 M MgCl_2_ and 0.5 M sucrose) was added and recovered at 37°C for 1 h. Subsequently, the cells were plated on MRS plates containing 5 μg/mL chloramphenicol and erythromycin. The expected recombinants were verified by PCR with the appropriate checking primers. For excision of the *cat* selectable marker, the expected recombinants were cultured on LMRS at 37°C with 5 μg/mL erythromycin for 24 h, and then streaked on a LMRS plate containing 2% lactose with 5 μg/mL erythromycin. After single colony isolation, the deletion mutants were tested by PCR with the appropriate checking primers.

### The Single-Plasmid Genome Editing System for Iterative Gene Knockout in *L. casei* BL23

To demonstrate the single-plasmid genome editing system for iterative gene knockout in *L. casei* BL23, *pflB, ldh* and *pdhC* responsible for acetoin biosynthesis were deleted sequentially as described above. The donor strains were listed in Table 3. To cure the plasmid pMSP456-Cre, the final mutants with desired deletions should be cultured at 37°C without erythromycin for 24 h and streaked on an MRS plate. After single colony isolation, the plasmid free mutant would be obtained.

**Table 3:** Experimental details for the single-plasmid system.

Strains designation	Target gene	Donor strains	Length of the deleted region (bp)	Efficiency^a^	
				
				Recombineering	Marker excision
BL24	*hicD3*	BL23	906	2/2	6/10
BL25	*pflB*	BL24	2262	2/2	6/10
BL26	*ldh*	BL25	981	2/2	4/10
BL27	*pdhC*	BL26	1665	2/2	6/10


*^a^“m/n” indicated that “n” colonies were randomly selected for screening and “m” colonies were demonstrated to be correct.*

### Fermentation Conditions and Analytical Methods

The engineered strains were pre-cultured (2% v/v inoculum) in 10 mL MRS medium for 18 h statically, and 4 mL of the above cultures were incubated in 200 mL MRS medium under shaking (baffled shaken flasks on a rotary shakers at 200 rpm) or static conditions at 37°C. Glucose, lactate, ethanol, acetate, and formate contents were measured according to Guo et al. (2012). Briefly, the concentration of these metabolites were analyzed by high-performance liquid chromatography (HPLC; Shimazu, Japan) using a column of Aminex HPX-87H Ion Exclusion particles (300 mm × 7.8 mm; Bio-Rad) at 55°C with a refractive index detector (RID). The mobile phase was 5 mM sulphuric acid at a flow rate of 0.4 mL min^-1^. Acetoin was determined according to Benson et al. (1996) with a small modification. Briefly, a 40 μl sample of fermentation broth and 80 μl 1 M NaOH were heated at 44°C for 30 min. The reaction solutions were then made up to 1 mL with H_2_O. 60 μl above solutions were taken out and placed to a tube with 440 μl H_2_O, 100 μl 0.5% creative and 100 μl 5% α-naphthol. The tube was incubated at 20°C for 1 h before the absorption was read at 525 nm using an ultraviolet visible light spectrophotometer (PERSEE TU-1810; China).

### Statistical Analysis

Statistical analysis was performed using unpaired two-tailed Student’s *t*-tests. *P* values of < 0.05 were considered statistically significant. *P*-values of < 0.01 were considered statistically high significant.

## Results

### Demonstration the Feasibility of the Single-Plasmid Genome Engineering in *L. casei* BL23

**FIGURE 2:**
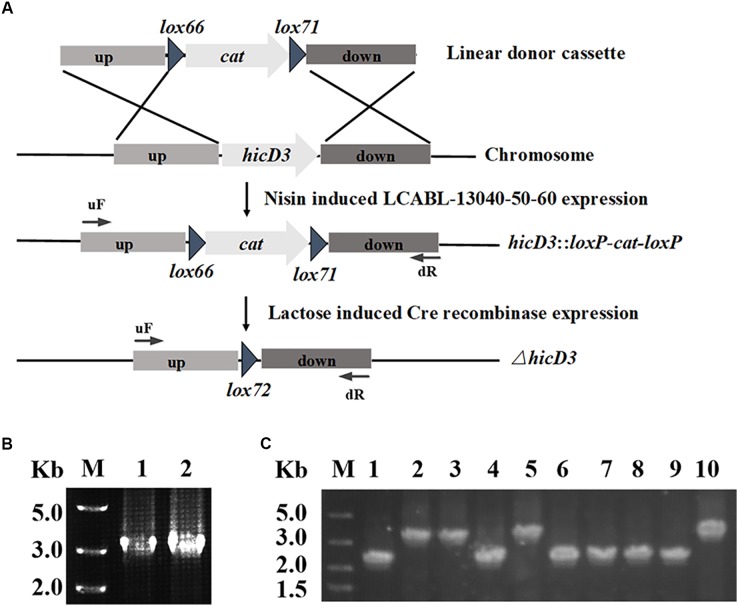
Scheme for one gene deletion using the single-plasmid system in *L. casei* BL23. **(A)** Diagram showing in-frame *hicD3* deletion and *cat* marker excision. Nisin induced LCABL_13040-50-60 mediated allelic replacement resulted in disruption of the *hicD3* gene and insertion of the *cat* marker, while lactose induced Cre subsequently excised the marker. **(B)** Two single colonies were randomly picked and verified by PCR with the forward primer of the up homology (uF) and the reverse primer of the down homology (dR). The PCR fragment of the strain in which the *cat* gene has replaced the *hicD3* gene was about 3.0 kb. **(C)** Ten colonies were randomly selected to tested by PCR with the forward primer of the up homology (uF) and the reverse primer of the down homology (dR) after Cre recombinase induction and incubation. The expected PCR fragment from the excision of the *cat* fragment was about 2.0 kb while the wild-type PCR fragment was about 3.0 kb.

Using the plasmid pMSP456-Cre (Figure 1A), a single-plasmid genome editing system was developed for markerless gene(s) deletion in *L. casei* BL23 (Figure 2A). The plasmid pMSP456-Cre was electroporated into the *L. casei* BL23 with an efficiency of ∼2.5 × 10^4^ CFU/μg plasmid DNA. To demonstrate the feasibility of this system, the gene *hicD3* (GenBank: CAQ67824.1) encoding for L-2-hydroxyisocaproate dehydrogenase was used as a target gene, expectedly, it was deleted successfully, yielding mutant *L. casei* BL24. Firstly, the nisin inducible expressed recombinases LCABL_13040-50-60 were used to targeted incorporate double-stranded DNA donor cassette (up-*lox66*-*cat*-*lox71*-down) into the genome of *L. casei* BL23, namely the recombineering step. After electroporation of 4 μg linear DNA donor cassette (up-*lox66*-*cat*-*lox71*-down) designed for *hicD3* gene, ∼200 CFUs were obtained. As shown in Figure 2B and Table 3, the randomly selected two recombinants were both verified to be the expect mutants by PCR. Secondly, to achieve markerless gene(s) deletion, the lactose inducible expressed site-specific recombinase Cre was employed to excise chloramphenicol resistant gene *cat* and only left a *lox72* site on the chromosome. After lactose induction, ten colonies were selected randomly and verified by PCR. As shown in Figure 2C, the efficiency of the marker excision was 60% for *hicD3* deletion.

### Scheme for Iterative Gene Deletion in *L. casei* Using Plasmid pMSP456-Cre

**FIGURE 3:**
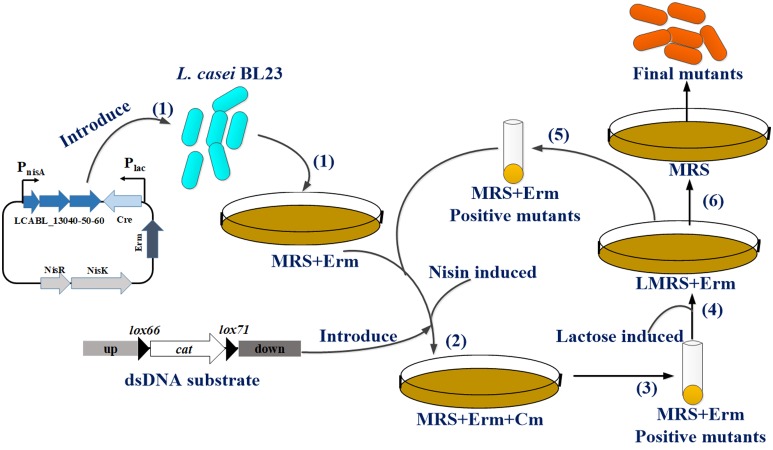
Overall scheme for iterative gene deletions using the single-plasmid system in *L. casei* BL23. **(1)** Plasmid pMSP456-Cre is first loaded into *L. casei* BL23 cells and selected the recombinants by MRS plates supplemented with 5 μg/mL erythromycin (Erm). **(2)** *L. casei* BL23 or derivates harboring plasmid pMSP456-Cre are made competent for electroporation of dsDNA substrate (up-*lox66-cat-lox71*-down) after induced with nisin to expression prophages LCABL_13040-50-60 and selected by MRS plates supplemented with 5 μg/mL Erm and chloramphenicol (Cm). **(3)** Recombinants are grown on Erm/Cm plates and positive mutants are verified by PCR with appropriate primers. **(4)** The positive mutants are grown on LMRS plates with 5 μg/mL Erm to induce the Cre recombinase expression for *cat* (chloramphenicol resistant gene) excision. **(5)** Positive mutants were also picked and verified by PCR and went to step **(2)** for sequential deletions or step **(6)** to obtain the final mutants could grow on MRS without selection to cure the plasmid pMSP456-Cre to terminate the cycle.

**FIGURE 4:**
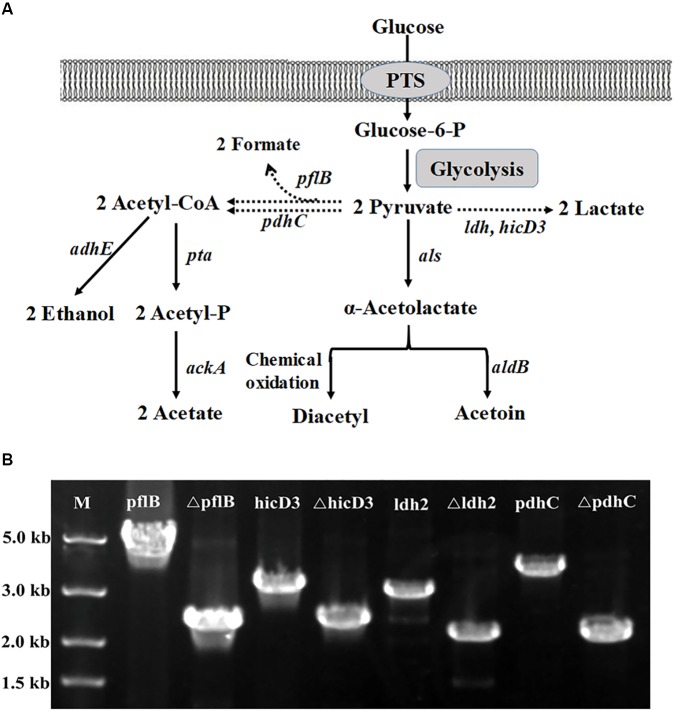
Metabolic pathways associated with acetoin biosynthesis in *L. casei*. **(A)** The metabolic engineering strategies for aerobic acetoin overproduction. The dashed arrows indicated that the pathways were disrupted. *ldh*, encoding Lactate dehydrogenase; *als*, encoding α-acetolactate synthase; *aldB*, encoding acetolactate decarboxylase; *pdhC*, encoding pyruvate dehydrogenase complex E2 component; *pflB*, encoding pyruvate formate lyase; *adhE*, encoding alcohol dehydrogenase; *pta*: encoding phosphate acetyltransferase; *ackA*: encoding acetate kinase. PTS, phosphotransferase system. **(B)** One colony of the BL27 strain was selected to be tested by PCR with the forward primer of the up homology and the reverse primer of the down homology after Cre recombinase induction and incubation. The expected PCR fragment from the deletion type is about 2.0 kb, while the wild-type is approximately 5.0 kb for *pflB* and 3.0 kb for *hicD3, ldh, pdhC*.

To further extend the pMSP456-Cre vector for multiple gene knockouts which requires iterative editing of the genome, a two-step procedure was employed for iterative and markerless gene(s) deletion in *L. casei* BL23 (Figure 3). The genes involved in pyruvate metabolism, including *pflB* (GenBank: CAQ66715.1) encoding formate acetyltransferase, *ldh* (GenBank: CAQ67767.1) encoding L-lactate dehydrogenase and *pdhC* (GenBank: CAQ66619.1) encoding pyruvate dehydrogenase complex E2 component, were sequentially deleted, yielding three mutant strains *L. casei* BL25, BL26 and BL27 (Figures 4A,B and Supplementary Figure S1). The efficiency of the single-plasmid system was shown in Table 3. The efficiencies of the marker excision step for those genes were 40∼60%.

### Acetoin Production of the Engineered Strains

**FIGURE 5:**
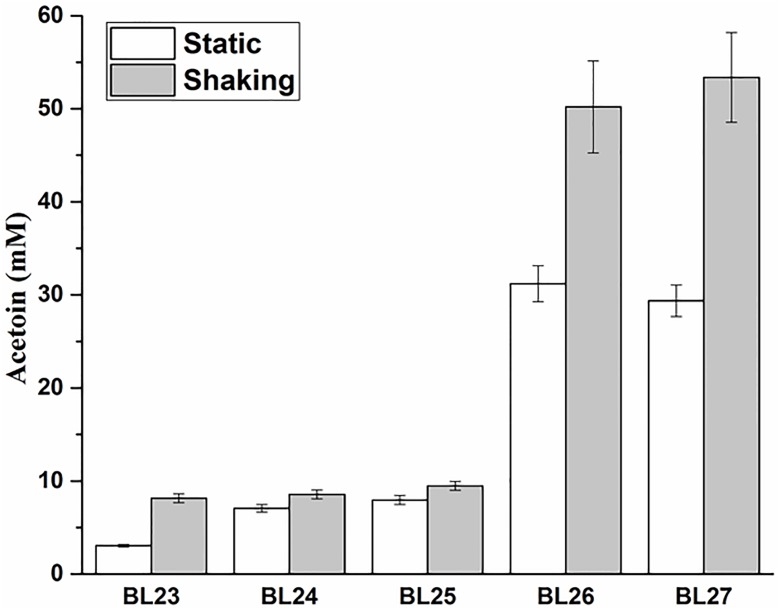
The acetoin production in strains BL23, BL24, BL25, BL26, and BL27 grown under shaking and static conditions for 24 h. Results are the averages from three independent experiments with standard deviations indicated by error bars.

**FIGURE 6:**
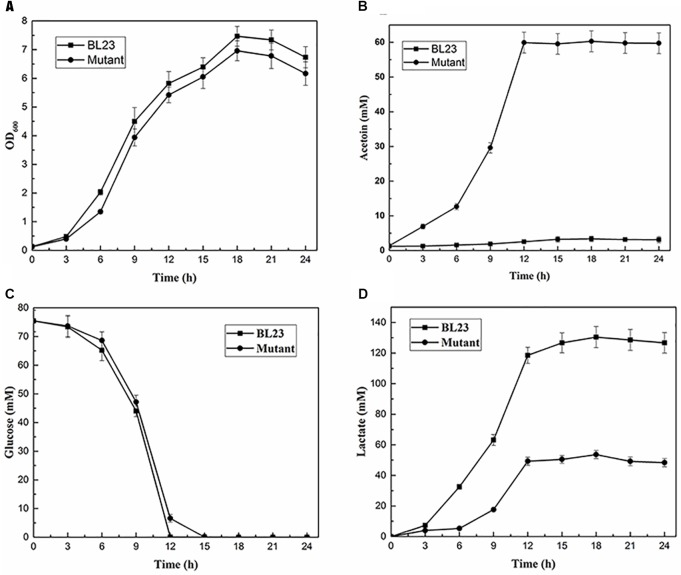
Fermentation profiles of the quadruple deletion mutant compared to the wild-type BL23 in MRS medium under shaking conditions. **(A)** Cell growth; **(B)** Acetoin production; **(C)** Glucose consumption; **(D)** Lactate production. Results are the averages from three independent experiments with standard deviations indicated by error bars.

The above four deletion mutants were cultured at 37°C under static and shaking conditions for 24 h and the acetoin production were measured. The results showed that the triple and quadruple mutants *L. casei* BL26 and BL27 could produce the same yield of acetoin (*P* > 0.05) under shaking condition (Figure 5). For further studies, we still selected the quadruple mutant *L. casei* BL27. As shown in Figure 6A, the growth rate of quadruple deletion mutant was slightly slower and reached a maximum OD_600_ of about 93.1–94.6% of the wild-type strain. However, Figure 6B shows ∼18-fold more acetoin production for BL23 relatively to the mutant (3.25 ± 0.12 to 57.98 ± 3.05 at 15 h) and this difference is stable between 12 and 24 h of the culture. For Figure 6C, the two curves of glucose consumption are almost superimposable. The only one obvious difference is that glucose is completely depleted after 12 h culture in BL23 strain while its total depletion happens after 15 h in BL27 mutant. Figure 6D shows a lower lactate production for BL27 mutant than the wild-type strain BL23. The acetate production of the BL27 strain was higher than that of the wild-type BL23 (Supplementary Figure S2A). Only a little ethanol was observed in the mutants while no ethanol was detected in the wild-type strain *L. casei* BL23 (Supplementary Figure S2B), and no formate production was detected under the fermentation conditions used in this study (data not shown).

## Discussion

In recent years, metabolic engineering has been beneficial to the production of many valuable metabolites and biochemicals in food and medicinal plants (Wilson and Roberts, 2014; Cho et al., 2017). However, it is still time-consuming and laborious to engineering a designed *L. casei* strain for production of the desired metabolites. In this study, we proposed a novel single-plasmid system for sequentially generating combinatorial markerless chromosomal deletions in *L. casei* BL23. To our knowledge, this is the first time to introduce the recombineering system and the Cre-*lox* system into the same vector for simplified and programmable construction of chromosomal deletions in *L. casei* BL23.

For markerless deletion of a targeted region of the *L. casei* BL23, the laborious and inefficient vector-based double-crossover strategies have been employed (Song et al., 2014). Currently, there is an increasing interest of using the CRISPR/Cas9 or CRISPR-Cas9^D10A^ system for gene deletions in bacteria, including *L. casei* (Song et al., 2017). The efficiencies of in-frame deletions were 25–62% (Song et al., 2017), corresponding to the efficiency of our system (40–60%). However, for sequential gene deletions, the recombinants containing editing plasmids need to be streaked on MRS plates without erythromycin for about 2–3 times to cure the plasmid (Song et al., 2017). In our previous study, a two-plasmids (pMSP456 and pMSPCre) based genome editing tool have been established for construction of deletion mutants with an efficiency of ∼100% (Xin et al., 2017). However, it is too time-consuming to cure these two plasmids and retransformation of them for sequential genes deletion (Xin et al., 2017). Deletion of a gene need ∼12 days using the two-plasmids based genome editing system, longer than that of the developed single-plasmid system in this study (only 7 days). Therefore, in this study, the new single-plasmid genome engineering strategy was established to address the problem of the time-consuming plasmid curing process in sequential deletions and a series of mutants for acetoin production have been constructed using this system. However, the yields of the acetoin of the mutant BL27 were lower than that of the engineered *L. lactis* strain (Liu et al., 2016b). Further studies should be down to improve the production of acetoin, such as increasing ATP demand or optimization the NAD^+^/NADH ration (Sun et al., 2012; Liu et al., 2016a).

In this deletion procedure, expression of the Cre recombinase gene was under the control of the tightly regulated promoter P_lac_ and the glucose and lactose simultaneously added to the culture would completely inactive the promoter P_lac_ (Alpert and Siebers, 1997). Therefore, to induction of the Cre recombinase, the lactose could be added solely to the sugar-free culture. To maintain the growth of the strains, the *L. casei* strains should be able to metabolize and deplete the lactose. Fortunately, previous studies have reported that only the ribose in the sugar-free culture could also active the promoter P_lac_ which was also demonstrated in our study (Gosalbes et al., 1999). Using the ribose as a carbon resource, we also demonstrated that this single-plasmid system could be applied in *L. casei* str. Zhang with an efficiency of ∼20% which could not metabolize lactose (data not shown). The reason of the lower efficiency was ribose does not act as an inducer in this case. Because LacT was not presented in *L. casei* str. Zhang, LacT cannot mediate antitermination, resulting in the expression of Cre recombinase under the control of the lac promoter was only regulated by carbon catabolite repression (CCR) (very low expression in glucose and moderate expression in ribose). Moreover, CCR is very low with ribose compared to glucose, resulting in a higher transcription rate from the lac promoter and a moderate expression of Cre which was functional for excision the *cat* gene with a low efficiency.

Moreover, due to the expression of the lactose operon in *L. casei* BL23 is subject to dual regulation: CCR and induction by lactose through transcriptional antitermination, the basal expression level of the P_lac_ promoter was very low in the absence of the inducer (Alpert and Siebers, 1997; Monedero et al., 1997; Gosalbes et al., 1999). This allows us to tightly regulate the Cre recombinase expression by P_lac_ during the replacement of the targeted gene with the dsDNA substrate cassette (up-*lox66-cat-lox71*-down), thus minimizing the unwanted cleavage of the *cat* gene and resulting in increased recombineering efficiency. However, the lactose-inducible promoter which was regulated by the LacT antiterminator would also limit its use in other lactobacilli strains. Therefore, further studies should be focused on the development the novel commonly widespread inducible system, like NICE system in LAB.

In this study, two main lactate dehydrogenase gene (*ldh* and *hicD3*) were deleted and it still produced a measurable amount of lactate which was similar to the previous reports (Rico et al., 2008). This could be explained that the other low activity lactate dehydrogenase gene is enough to give rise to a substantial lactate accumulation in the fermentation broth (Rico et al., 2008). Moreover, deletion of *ldh* and *hicD3* would result in the accumulation of pyruvate which could be metabolized to malate by malate dehydrogenase. Afterward, the malate could be degraded rapidly to the lactate by the malolactic enzyme (Landete et al., 2013). Moreover, though deletion of *pdhC* and *pflB* genes, low yields of ethanol were still detected in BL27 strain while no ethanol was detected in wild-type BL23 strain even the ethanol would be evaporated at 37°C under shaking conditions. We think in wild-type strain, the NAD^+^/NADH ratio was in balance while deletion of the major *ldh* gene would result in the accumulation of the NADH and pyruvate. In general, the accumulated pyruvate would be oxidized to acetyl-P by pyruvate oxidase. Acetyl-P would generate acetyl-CoA through the phosphate acetyltransferase, and then to balance the NAD^+^/NADH ratio, the very low yields of the ethanol would be generated from acetyl-CoA by acetaldehyde dehydrogenase/alcohol dehydrogenase. In wild-type, the acetate was detected because of the sodium acetate added in the MRS medium. However, low yields of acetate could be produced in BL27 strain. We think the acetate was produced from the citrate metabolism (Palles et al., 1998) because the citrate from the diammonium citrate added to MRS medium could be completely degraded after 12 h in BL27 mutant while the amounts of citrate were unchanged during the culture process in BL23 strain (data not shown).

## Conclusion

In this study, we achieved the successfulness of the sequential construction of mutants using this tool in *L. casei* BL23. We expected that this single-plasmid system could provide a new way for metabolic engineering and generation reconstructed *L. casei* strains served as cell factories for production high-valued metabolites. Further studies should be focused on the development of novel commonly widespread inducible systems, like NICE system, and exploring the universal prophage recombinases to extend its use in other bacteria of interest.

## Author Contributions

YX and JK designed the study. YX conducted the experiments. YX, TG, and YM contributed to the experimental design and data analysis. YX, TG, YM, and JK wrote the paper.

## Conflict of Interest Statement

The authors declare that the research was conducted in the absence of any commercial or financial relationships that could be construed as a potential conflict of interest.
