# Supplementary material for: A Single-Plasmid Genome Editing System for Metabolic Engineering of Lactobacillus casei
Source: Front Microbiol. 2018 Dec 5;9:3024. doi: 10.3389/fmicb.2018.03024 (PMC6289983; doi:10.3389/fmicb.2018.03024)
Supplement: Supplementary file 1 [file Data_Sheet_1.pdf]

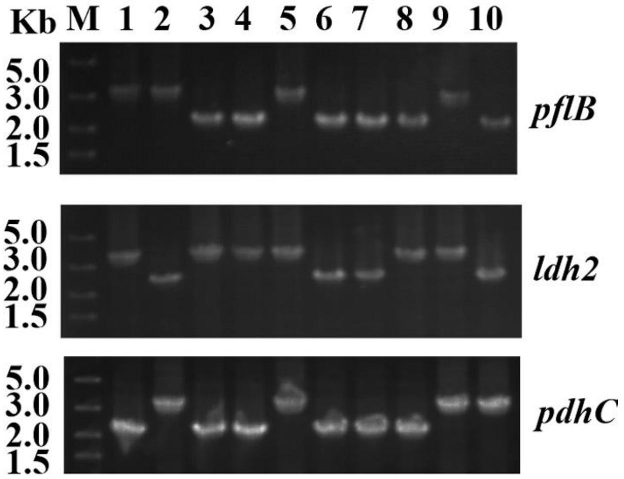

FIG S1 Ten colonies from every deletion were randomly selected to tested by PCR with the forward primer of the up homology and the reverse primer of the down homology after Cre recombinase induction and incubation. The expected PCR fragment from the deletion type was about 2.0 kb, while the wild type PCR fragment was approximately 3.0 kb.

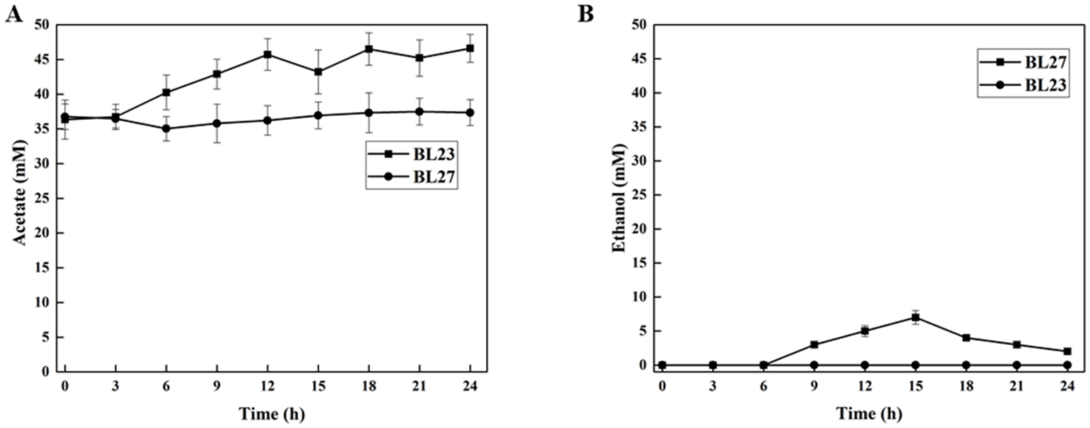

FIG S2 Fermentation profiles of the quadruple deletion mutant compared to the wild-type BL23 in MRS medium under shaking conditions. (A) acetate and (B) ethanol. Results are the averages from three independent experiments with standard deviations indicated by error bars.
